# JCVI: A versatile toolkit for comparative genomics analysis

**DOI:** 10.1002/imt2.211

**Published:** 2024-06-12

**Authors:** Haibao Tang, Vivek Krishnakumar, Xiaofei Zeng, Zhougeng Xu, Adam Taranto, Johnathan S. Lomas, Yixing Zhang, Yumin Huang, Yibin Wang, Won Cheol Yim, Jisen Zhang, Xingtan Zhang

**Affiliations:** ^1^ Fujian Provincial Key Laboratory of Haixia Applied Plant Systems Biology, Haixia Institute of Science and Technology and College of Life Sciences Fujian Agriculture and Forestry University Fuzhou Fujian China; ^2^ J. Craig Venter Institute Rockville Maryland USA; ^3^ National Key Laboratory for Tropical Crop Breeding, Shenzhen Branch, Guangdong Laboratory for Lingnan Modern Agriculture, Genome Analysis Laboratory of the Ministry of Agriculture, Agricultural Genomics Institute at Shenzhen Chinese Academy of Agricultural Sciences Shenzhen Guangdong China; ^4^ National Key Laboratory of Plant Molecular Genetics (NKLPMG), CAS Center for Excellence in Molecular Plant Sciences (CEMPS), Institute of Plant Physiology and Ecology (SIPPE) Chinese Academy of Sciences (CAS) Shanghai China; ^5^ School of BioSciences The University of Melbourne Melbourne Victoria Australia; ^6^ Department of Biochemistry and Molecular Biology University of Nevada Reno Nevada USA; ^7^ State Key Lab for Conservation and Utilization of Subtropical Agro‐Biological Resources, Guangxi Key Lab for Sugarcane Biology Guangxi University Nanning Guangxi China

**Keywords:** comparative genomics, genome annotation, genome assembly, genomic data, visualization

## Abstract

The life cycle of genome builds spans interlocking pillars of assembly, annotation, and comparative genomics to drive biological insights. While tools exist to address each pillar separately, there is a growing need for tools to integrate different pillars of a genome project holistically. For example, comparative approaches can provide quality control of assembly or annotation; genome assembly, in turn, can help to identify artifacts that may complicate the interpretation of genome comparisons. The JCVI library is a versatile Python‐based library that offers a suite of tools that excel across these pillars. Featuring a modular design, the JCVI library provides high‐level utilities for tasks such as format parsing, graphics generation, and manipulation of genome assemblies and annotations. Supporting genomics algorithms like MCscan and ALLMAPS are widely employed in building genome releases, producing publication‐ready figures for quality assessment and evolutionary inference. Developed and maintained collaboratively, the JCVI library emphasizes quality and reusability.

## Highlights

- JCVI is a Python‐based library that enables genomic workflows through a collection of simple reusable tools.
- The JCVI library is modular with basic functionalities separated into bioinformatics format parsing, assembly and annotation‐related tools, comparative genomics, and graphics generation.
- Embedded algorithms like MCscan, ALLMAPS, and other tools within JCVI are now widely used in the community and power a wide array of use cases.

## Graphical abstract

The JCVI library contains a set of computational tools that are often used in tasks covering genome assembly, annotation, and comparative genomics. Engineered with a focus on versatility, the library incorporates modules for algorithms, format parsing, and graphics generation, enabling seamless integration into diverse research workflows.

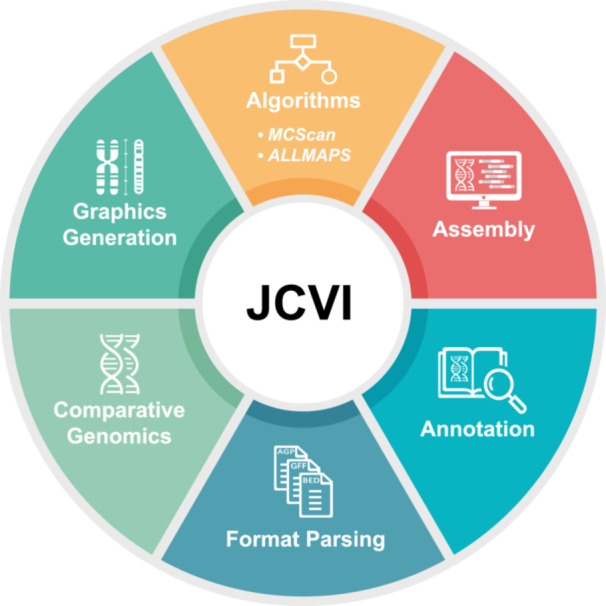

## INTRODUCTION

In the dynamically evolving field of genomics, genome assembly, annotation, and comparative genomics have become foundational processes that enable researchers to decipher the code of life [1, 2, 3, 4]. Recent research not only provides insights into genetic variation and evolutionary relationships but also empowers the scientific community by providing the tools to address ongoing challenges in health, agriculture, and biodiversity conservation [5, 6]. The advent of high‐throughput sequencing technology, coupled with democratization in bioinformatics tools, has ushered in an era of high‐quality genome assemblies with increased power and precision. These advances now fuel an unprecedented rate of discovery in evolution and biodiversity as projects to sequence thousands of species are underway [7].

With the explosive growth of genomic data, the field of bioinformatics has also become increasingly sophisticated, due to advancements in sequencing technology. Such sophistication is enabled by the increased uptake of scripting languages (e.g., Python, Perl, and R) to rapidly prototype bioinformatics algorithms and workflows. For example, TBtools [8] is a popular software designed to offer a user‐friendly bioinformatics platform mostly focused on analyses of functional genomics data, including RNA‐seq, small‐scale sequence alignment, and evolutionary analysis. Although more than 50 functional plugins have been developed, TBtools's mostly static graphical user interface (GUI) setup can occasionally restrict users from further customizations. Python has been consistently ranked first in terms of popularity on the TIOBE index (TIOBE Software BV) as of March 2024. The popularity of Python owes to its clean syntax, readability, and expressiveness, which makes it ideal for scientists delving into computational work. Python has a rich ecosystem of third‐party libraries to tackle various complex bioinformatics challenges. Python also owes its success to the versatile array programming interface provided by libraries like NumPy, SciPy, and PyTorch that facilitate writing performant code [9] and enable a natural way of handling large data sets that are even non‐numeric and of variable length, such as DNA sequences [10].

Python libraries for bioinformatics have existed for over 20 years, such as Biopython [11], which provide tools for developing genomics pipelines. Biopython offers low‐level capabilities for reading and writing different sequence or alignment file formats, interacting with databases of biological data, conducting basic sequence analysis, and driving command‐line tools. Biopython not only facilitates the development of genomic pipelines but also ensures that such pipelines are robust, reproducible, and adapted to the evolving landscape of genomics research. This model has influenced the design of later libraries including JCVI.

Despite the availability of Biopython, which provides some building blocks for bioinformatics, the complexity of genomic data often calls for specialized, high‐level tools that can address the challenges of genome assembly and comparative genomics that are often more complex [12]. Specifically, evaluation and comparisons between genome assemblies and annotations are routinely performed. Synteny analysis has also become a foundational framework of comparative genomics, where the conservation of homologous genes and gene order is identified between genomes of different species [13]. Visual representations are instrumental in synteny analysis that aids in both identifying and verifying conserved gene orders [13, 14]. The WGDI package offers functions for comparative genomics, such as synteny analysis and whole‐genome duplication [15]. While other tools exist in each of these spaces, there is a demand for tools that are designed from the ground up to integrate heterogeneous data from genome assembly, annotation, and comparative genomics that are pillars for modern genomic studies.

The development of JCVI was motivated by such unmet needs in the genomics field for a high‐level library to enable genome builds, comparisons, quality control, and concordance evaluation between multiple genetic maps. Traditional workflows often require researchers to navigate through a maze of separate applications for different aspects of comparative genomics, such as assembly alignment, synteny analysis, and visualization [16]. JCVI's integrated suite of tools and applications offers a unified turn‐key solution that simplifies these tasks, making it easier for researchers to conduct comprehensive genomics analyses in a streamlined fashion.

The JCVI codebase can be accessed from GitHub: https://github.com/tanghaibao/jcvi with detailed documentation on usage and examples. As of May 2024, the repository has gained 693 stars and 179 forks, which makes it among one of the most popular bioinformatics libraries on GitHub. The repository has been actively maintained over the 14 years of its existence, with over 400 community issues resolved. The source code of the toolkit can be downloaded and compiled locally or installed from prebuilt wheel packages available through PyPI: https://pypi.org/project/jcvi. To date, the JCVI toolkit has been downloaded over 220,000 times.

## RESULTS

### Overview of JCVI

The JCVI toolkit is designed to streamline genomic data manipulation and analysis, leveraging the versatility of Python across a wide range of bioinformatics tasks. It encompasses a broad spectrum of functionalities, from handling bioinformatics file formats like FASTA, FASTQ, AGP, BLAST, GFF3, and BED, and so on, to performing statistical summary, data extraction, conversion between these formats, or serving as a foundation for high‐level applications. As one example, the package's “formats.fasta” module specifically caters to FASTA formatted sequence data, offering capabilities for cleaning sequences, comparing records, extracting sequences by identifiers, and more. The module covers actions such as filtering sequences by size, generating gap size reports, analyzing G+C content distributions, and translating coding sequences to proteins. Parsers of frequently accessed formats, such as basic local alignment search tool (BLAST) [17, 18] tabular formats, are implemented in Cython [19] for improved performance (>2× speedup) compared to a pure Python implementation.

Beyond format manipulation, the JCVI suite excels in the visualization and analysis of genomic information. Through its “graphics” module and associated scripts, users can engage in detailed visualization tasks such as generating coverage histograms, creating gene and sequence features, visualizing chromosome karyotypes, and plotting assembly graphs and genomic maps. This suite enriches genomic research through intricate representations of genomic alignments and assembly quality assessment.

The JCVI suite further contains high‐level modules that go beyond simple format parsing and graphics generation capabilities. Most notable features include the “compara” module for comparative genomics analysis, offering tools for synteny block reconstruction [20], gene loss cataloging [21], quota‐based synteny alignment [22], pedigree visualization [23], and *K*_s_ calculations and visualizations, among others. This module is crucial for exploring evolutionary relationships and genome architecture across species. Additionally, the “assembly” module facilitates genome assembly and mapping tasks, such as constructing pseudo‐chromosome sequences, estimating gap sizes, merging genetic maps into a consensus sequence [24], and comparing against genetic and Hi‐C contact maps, providing tools for researchers aiming to achieve high‐quality genome assemblies.

Other useful modules include “annotation” to handle gene annotations, naming, and quality control, particularly useful when building genome releases. The “annotation” module mostly contains scripts to drive a comprehensive annotation pipeline. The driver scripts are able to control the life cycle of an annotation project, including the training of ab initio gene predictors, postprocessing of structural annotations (e.g., MAKER [25]), and functional annotations (e.g., AHRD [26]). The “apps” module contains utilities to communicate with GenBank, Short Read Archive (SRA), and Phytozome [27], as well as a wrapper for common bioinformatics applications.

Taken together, these modules within the JCVI package underscore its importance in bioinformatics, offering an accessible, flexible, and powerful resource for scientists to address complex genomic data analysis and visualization challenges.

### Case studies

As follows, we show several high‐level use cases for the JCVI library. Details of the use cases can also be accessed from the wiki pages on GitHub: https://github.com/tanghaibao/jcvi/wiki, with tutorials and hosted test data to guide users through examples.

### Case I: Synteny inference and visualization with MCscan

MCscan is a computational tool implemented in the JCVI package (within module “compara”) and has become a popular choice in the field of bioinformatics and genomics for the analysis of synteny and collinearity among multiple genomes [20, 28]. Synteny refers to the conservation of blocks of order within two or more genomes, which can provide insights into evolutionary processes, gene function, and the architecture of genomes [20]. MCscan leverages this concept by utilizing gene order and similarity to detect synteny blocks between multiple genomes, thereby facilitating comparative genomics studies.

The core principle behind MCscan is the identification of syntenic regions across different species (from orthology) or within species (from duplications) by comparing the order and sequence similarities between genes. This involves examining patterns of annotated genes from genome assemblies to identify regions with sequence conservation [29]. MCscan is particularly valuable in plant genomics, where frequent genome duplications may obscure evolutionary history. Some genome duplication events may occur before or after species divergence, creating multiple‐to‐multiple homologous regions as synteny blocks. The identification of synteny blocks therefore enables researchers to date the duplications, trace gene origins (e.g., genome duplicates, tandem duplicates, or single transpositions), and explore karyotype conservation.

**Figure 1:**
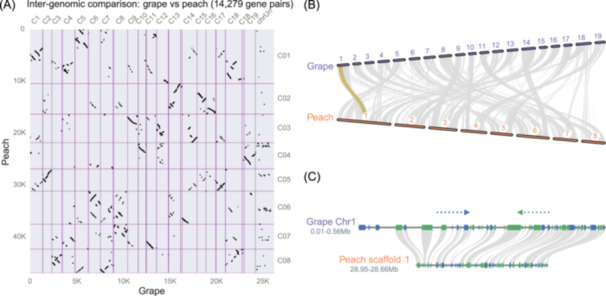
Genome dotplot, macro‐synteny, and micro‐synteny analyses between peach and grape genomes. (A) Dot plot of 14,279 gene pairs within synteny blocks resulting from the alignment of coding sequences between the peach and grape genomes. Counting the number of synteny blocks horizontally and vertically reveals a 3:3 pattern, suggesting that grapes and peaches share a genome triplication event. (B) Another illustration of synteny regions is the karyotype figure, which displays links between the chromosomes to show the gene pairs within synteny blocks. Users have the option to rotate the chromosomes for customized layout or highlight the genomic regions of interest that contain specific gene pairs (yellow). (C) Micro‐synteny analysis between a grape region and matching region in peach. The connecting ribbons show matching genes, with blue boxes showing genes on the positive strand, and green boxes showing genes on the negative strand.

As a concrete example, we analyzed grape and peach genomes sourced from the Phytozome database [27]. Following the detection of synteny blocks, MCscan generates graphical representations such as dot plots and chromosomal maps, which illustrate the syntenic relationships across chromosomes. Interpreting the dot plot involves observing the number of blocks aligned horizontally or vertically. For example, each peach region correlates with up to three synteny regions in grape and vice versa (Figure 1A). This reflects the shared genome triplication (also known as γ‐event) evident in the 3:3 pattern observed [30]. Chromosomal maps offer an alternative visualization, delineating chromosome karyotypes and the interconnectivity of synteny blocks via gray ribbons displayed between parallel tracks (Figure 1B). Further customization of these figures is possible, allowing for enhanced visual distinction of specific blocks by color‐coding, adjusting chromosome orientations, and flexible layout of the genomes for comparison. Increasingly sophisticated usage of MCscan that involves more genomes with varying ploidy levels can be found in Chalhoub et al. [21].

Additionally, users may wish to illustrate local synteny patterns, or “micro‐synteny,” with the gray ribbons now connecting between matching genes, instead of chromosomal segments as seen in the “macro‐synteny” analysis. This can be useful in elucidating local details at the scale of tens of genes. Similarly to the macro‐synteny figure, certain matches can be highlighted to support user narratives (Figure 1C). Additional options are available to change the style of the syntenic connections, as well as the ability to overlay extra features and gene names on the plot to assist in target inspections.

### Case II: Map‐based assembly and alignment with ALLMAPS

The precise ordering and orientation (OO) of genomic scaffolds for chromosome reconstruction is a cornerstone of de novo genome assembly. This process is enhanced by the integration of diverse mapping techniques, each offering orthogonal evidence supporting the final assembly. ALLMAPS (within the “assembly.allmaps” module) computes scaffold orders that maximize collinearity with a collection of maps, including genetic, physical, or comparative maps, thereby improving the accuracy and integrity of chromosomal assemblies [24].

**Figure 2:**
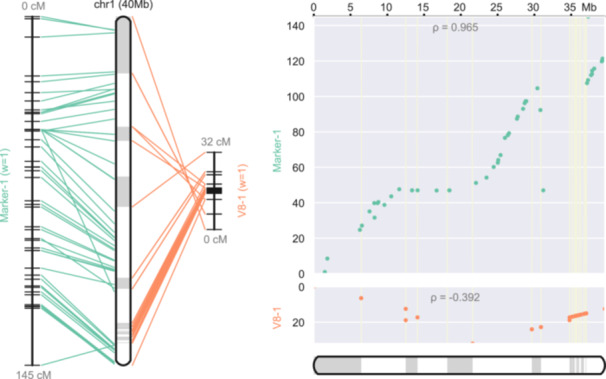
Pseudochromosome 1 of the *Carica papaya* genome, reconstructed from two input maps—an SSR‐based genetic map and a synteny map coordinated with a previously published papaya genome. The left panel presents connecting lines as matching markers. The right panel shows two sets of scatter plots with each dot representing the physical position on the chromosome (*x*‐axis) versus the map location (*y*‐axis). Spearman's ρ is displayed to indicate the concordance between the marker order on the chromosome with their map locations (between −1 and 1, with 1 being completely concordant).

The results generated by ALLMAPS are multifaceted, offering researchers both visual and statistical insights into genome assembly quality. Each reconstructed chromosome is reported as a separate portable document format (PDF) file (Figure 2). The side‐by‐side alignments in the left panel (the map view) facilitate the identification of conflicting markers through crossing lines, while the scatter plots on the right panel depict the physical and map locations of markers, assisting in visualizing collinearity and its disruptions. The accompanying summary report provides essential statistics including the number of scaffolds anchored, the total length of sequences, and so on, which are critical for evaluating the outcome of the assembly process.

ALLMAPS is a versatile tool with wide‐ranging applications in genomics and is particularly useful in plant genomics where polyploidy and high levels of genetic variation pose significant challenges to assembly. Through its capability to integrate multiple types of mapping data, ALLMAPS enhances our ability to construct accurate genome assemblies even in these complex scenarios. Additionally, its ability to split chimeric contigs and estimate gap lengths further contributes to its utility in refining genome assemblies.

### Case III: Genome features and landscape plotting

**Figure 3:**
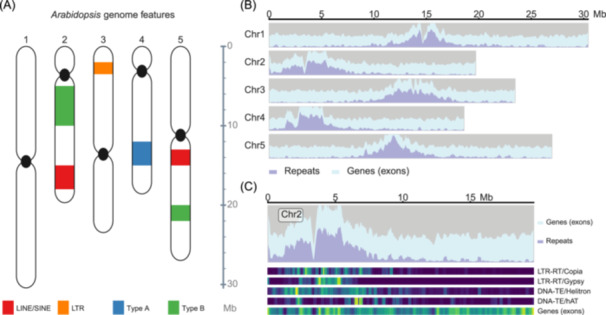
Examples of miscellaneous features visualized along the genome. (A) Chromosome painting with “graphics.chromosome” that shows the locations of various elements in the portrait mode. The chromosome sizes (scale on the right side of the chromosomes), location of the centromeres (filled ellipses in black), and genomic features (filled rectangles in various colors) can be specified as inputs to the script. (B) Genomic landscape plotted with “graphics.landscape.” The chromosome sizes, density of the gene, and repeat elements can be specified as inputs to the script and shown as stack plots. (C) Genomic landscape along a single chromosome, with alternative heatmap representations correlated with the feature density. Toy data are used for illustration purposes only. LTR, long‐terminal repeat.

JCVI contains numerous routines to create customized plots with the goal toward supporting tight data integration. In comparative genomics, custom visualization is often needed to overlay multiple dimensions of data on top of one another for exploratory analysis. Alternatively, we may wish to show different categories of features for several chromosome regions in a genome. For example, let us say a user wants to plot the Arabidopsis genome with certain genomic features overlaid on top. When supplied with some basic information, for example, the size of the chromosomes, the location of the genomic features (e.g., transposons, in the BED format), the “graphics.chromosome”module can be used to perform the plot. Features like centromeres are natively understood and can be readily plotted (Figure 3A).

Additionally, JCVI is capable of customizing genome landscape visualization, which is crucial in genome annotation. This includes the ability to create heatmaps that represent genome features alongside the genome. For instance, a user may want to visualize a genome or chromosome of interest with specific gene or transposon features using stack plots or heatmaps (Figure 3B,C). By providing basic information such as chromosome sizes and the locations of genomic features (e.g., transposons in the BED format), the “graphics.landscape” module can be employed to generate plots. Native support for features like transposon element classification allows for their straightforward inclusion in the visualizations (Figure 3B,C).

### Case IV: Genome‐build quality control

**Figure 4:**
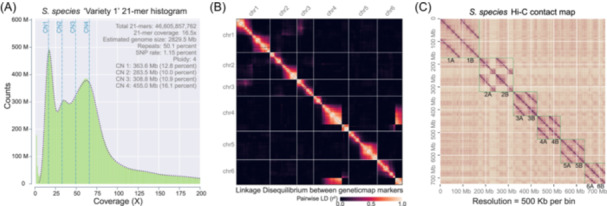
Examples of genome‐build quality control. (A) K‐mer spectrum analysis to estimate genome parameters based on raw sequencing data. (B) Concordance between genome assembly and the linkage map. (C) Concordance between genome assembly and the Hi‐C contact map. These are all powerful tools (“assembly.kmer,” “assembly.geneticmap,” and “assembly.hic”) to evaluate the quality of a genome build.

JCVI includes a suite of tools at all stages of genome assemblies, including a preassembly genome survey to postassembly quality control. The raw sequencing data can often be used to derive the basic statistics of a genome through the analysis of *K*‐mer spectra [31]. JCVI implements two separate methods for estimating the genome statistics: the negative binomial mixture model and the ALLPATHS method (see the Methods section). Specifically, the genome size, ploidy, heterozygosity, and repeat content can all be estimated on the basis of sequencing data alone, as part of the “assembly.kmer” module (Figure 4A).

After the draft genome assembly, there is a repeated need for quality control to ensure that the assembled genome is concordant with orthogonal evidence, including the genetic map and Hi‐C. JCVI can facilitate the evaluation of the genome assembly and linkage patterns (Figure 4B). Entries in the heatmap correspond to the pairwise linkage disequilibrium (*r* ^*2*^) where diagonal entries are mostly expected that indicate a tight linkage between physically close markers. Off‐diagonal linkage patterns, such as chr4 and chr6 in the example shown (Figure 4B), may suggest a potential misassembly. Similarly, heatmaps based on the Hi‐C contact map can also be a powerful tool to evaluate the quality of the genome build, with disruptions to the otherwise diagonal patterns suggestive of misassemblies (Figure 4C). These concordance analyses are wrapped as part of “assembly.geneticmap”and “assembly.hic”modules.

### Case V: Pedigree and genome diversity

**Figure 5:**
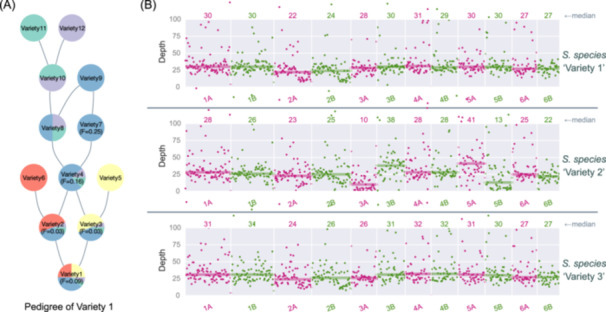
Examples of pedigree and genome diversity analysis. (A) Pedigree of a particular variety (Variety 1; at the bottom), as encoded in a PED file, can be visualized with the “compara.pedigree” module. The root nodes (nodes with no parent information at the top) are assumed to be outcrossing for simplicity. The parentage in each variety is then estimated and visualized as pie charts. *F*: inbreeding coefficients estimated based on the pedigree. (B) Copy number variation between different varieties as visualized with “graphics.landscape” module.

Our final use case illustrates tools in JCVI to help with the analysis of pedigree and genomic variation between varieties. These tools can be useful in re‐sequencing projects aiming at the study of genome diversity. One basic analysis is to visualize the pedigree of varieties that, for example, may illustrate breeding history if the genome in a study is a crop variety. JCVI can visualize a pedigree of varieties, based on the relationship encoded in the PED file [23], using the “compara.pedigree”module. We can estimate the parentage in the form of pie charts colored by the parental nodes along the pedigree (Figure 5A). The inbreeding coefficients (*F*) are also estimated to indicate the probability of two alleles in an individual, which are identical by descent from a common ancestor (Figure 5A).

In resequencing projects, it is often useful to visualize the genomic variation between varieties, for example, copy number variations (CNVs). In the given example, the sequencing depth patterns could reveal certain chromosome deletion events (e.g., chr3A) and duplications (e.g., chr3B), as shown in reduced or elevated sequencing depths, respectively (Figure 5B). This tool may reveal particularly useful genomic events during breeding or evolution [6]. The CNVs are only used here as a motivating example; other variant patterns or genomic features can be similarly plotted and contrasted to gain intuition.

### Command line usage

The command line usage of the JCVI toolkit is designed to be simple and introspective. All command line tools are conveniently organized into modules, scripts, and actions. The general usage pattern is

$ python ‐m jcvi.[module].[script] [action]

For example, the ALLMAPS tool is available under the “assembly” module, “allmaps” script. In the case when the user fails to identify the exact name of the script, the command line facilitates discovery by listing all available scripts under “assembly” with

$ python ‐m jcvi.assembly

All available actions under “allmaps” can then be listed with

$ python ‐m jcvi.assembly.allmaps

Finally, users can examine available arguments and options for specific action, such as “path.”

$ python ‐m jcvi.assembly.allmaps path

All other tools follow similar interfaces and usage patterns.

## DISCUSSION

JCVI's innovative approach lies in its comprehensive suite of tools designed for the intricate needs of comparative genomics. The JCVI library is modular, with utilities for bioinformatics format parsing, graphics generation, and exploratory tools linking the pillars of assembly, annotation, and comparative genomics that provide a robust framework for researchers. Embedded applications like MCscan for synteny inference and visualization [28], and ALLMAPS for map‐based assembly and alignment [24], exemplify JCVI's capability to address the complex yet common challenges in genomics. The primary mode that users interact with JCVI is through its simple command line interface (CLI), which differs from tools like TBtools [8], which provide mostly a GUI framework. More advanced use of JCVI requires programmatic access to its reusable components. This mode defines a unique tradeoff between the user learning curve and customizability.

Visualization emerges as a crucial element in genomic research, with JCVI providing powerful tools for synteny analysis, chromosome mapping, and other visual representations. Previous tools such as SyMap [32], Chromosome Visualization Tool [33], and VGSC2 [34] can visualize synteny and collinearity. Each of these tools has contributed to the field by enabling the exploration and mapping of genomic similarities and variations across different organisms. However, MCscan distinguishes itself by integrating these visual cues into a set of feature extraction and plotting tools that can be chained and configured to support comparisons at multiple scales ranging from genome‐wide to “zoomed‐in” local regions.

Previous synteny analysis software such as MCscanX [28] and WGDI [15] mainly use BLAST‐based ortholog prediction, which relies on *E*‐value scores without the additional process. Relying solely on *E*‐value scores for ortholog prediction can be problematic because *E*‐value is dependent on the size of the database, which means that the significance of an alignment could change in different comparisons. This can lead to aggregated errors across multiple tests, which can lead to misleading results [35]. Moreover, MCscanX still relies on BLAST, which is significantly slower and susceptible to repetitive hits. In contrast, the JCVI synteny inference is based on adaptive seeds via LAST [18] and adopts rigorous filtering based on *C*‐score and removal of proximal duplicates, thereby circumventing the difficulties with finding hard *E*‐value cutoffs as well as avoiding artifacts from repeats. Previous studies have independently confirmed that overall JCVI performed better than MCScanX [15]. WGDI, while effective in analyzing whole‐genome duplications, lacks capabilities for microsynteny visualization and multigenome comparison, limiting its application in comparative genomic studies that require detailed visual analysis of gene order conservation across multiple genomes. WGDI also tends to offer fewer options to customize the canvas layout, which is essential to render the comparisons between multiple chromosomal regions and genomes.

The challenges of assembling large, complex, repeat‐rich, polyploid, or highly heterozygous genomes can make independent genome assembly and map construction both prohibitively expensive and inadequate for generating high‐quality sequences [36, 37]. To overcome these obstacles, one approach, exemplified by earlier map‐based software including JoinMap, MSTMap, and R/qtl, capitalizes on the synergies between genome sequencing and linkage mapping [38, 39, 40]. ALLMAPS emerges as a particularly innovative tool to optimize the integration of sequencing data with genetic or physical maps and genomic synteny [41]. ALLMAPS employs a sophisticated approach to reconcile discrepancies between different data types, iteratively ordering and orienting scaffolds to reflect the most accurate arrangement possible based on the available maps. This process not only enhances the fidelity of the assembled genome but also significantly contributes to our understanding and interpretation of complex genomic structures.

The capability of JCVI covers Hi‐C and linkage mapping data in the current era of many “telomere‐to‐telomere” (T2T) genome assemblies where the quality of the assemblies is of paramount importance. The quality of genome assemblies can be improved by integrating our prior developments from ALLHIC [42], ALLMAPS [24], linkage mapping tools [39], and MCscan outputs and bringing these evidence all under the same framework, as shown in Case III. This framework positions JCVI as a viable alternative solely based on Hi‐C‐based scaffolding or synteny‐based methods alone. While tools like RagOO [11] and RagTag [43] are capable of processing synteny and Hi‐C data simultaneously, the combined use of ALLMAPS, ALLHIC, MCscan, and genome‐build quality control tools within JCVI provides a more thorough approach. The concordance checks between synteny analysis, Hi‐C data, and other linkage mapping data for assembling complex genomes are powerful and visually driven, as shown in Case IV. Therefore, the tight integration and cross‐referencing between the comparative, assembly, and annotation modules are a defining feature of JCVI.

Through case studies, including synteny inference with MCscan, map‐based assembly with ALLMAPS, and many more, we have demonstrated how JCVI facilitates complex algorithms and workflows. The ongoing development of JCVI, driven by community feedback and the latest genomic discoveries, promises to further enhance its functionality and applicability. This progression is expected to shed light on similar efforts and ventures within the bioinformatics community, including notable open‐source projects like Bioconductor and BioJS [44, 45]. This community‐driven approach has been pivotal in ensuring the library's quality and reusability, underscoring the collaborative nature of advancements in bioinformatics. Future updates might include optimizations for next‐generation sequencing technologies, expanded libraries for emerging bioinformatics challenges, and more user‐friendly interfaces to accommodate researchers with varying levels of computational expertise.

## CONCLUSION

In summary, the JCVI library is a versatile toolkit that integrates various functions, including format parsing, genome assembly and annotation manipulation, genomics algorithms, and graphics generation. The JCVI library provides a simple, reusable, and unified solution for analyzing and visualizing genomic data, thereby facilitating bioinformatics research in genomics.

## METHODS

### MCscan

We performed synteny searches to compare between genomes and corresponding chromosomal regions. To call syntenic blocks, we performed all‐against‐all LAST [18] and chained the LAST hits with a default distance cutoff of 20 genes, with a minimum requirement of four gene pairs per syntenic block.

The sequence similarity is calculated based on an important “*C*‐score,” which is based on a ratio that compares the LAST score of a particular alignment to the highest score achieved by each sequence in any of their alignments.

$$C\mathrm{\_score}\left(A,B\right)=\frac{\mathrm{score}(A,B)}{\max\;(\text{best score for}\;A,\;\text{best score for}\;B)}.$$

The *C*‐score is the ratio of the LAST result score to the maximum score observed for either the query or subject involved in each LAST hit. This normalizes the score to a range between 0 and 1, where 1 would indicate that the hit has a score equal to the best score that the sequence has achieved in any LAST hit. The default *C*‐score cutoff is 0.7, representing a balance between finding orthologs and shared paralogs. Additionally, the gene pairs are filtered with respect to repetitive matches between proximal duplicates (default: within 10‐gene distance), and only the best scoring pair is kept as the representative pair during the initial seeding of the syntenic blocks.

The above parameters are all configurable on the command line. All computational steps, including drivers for LAST executions, are implemented in JCVI, as a single pipeline (“compara.catalog”).

### ALLMAPS

The principal challenge in genome assembly lies in the accurate OO of scaffolds, a task that is NP‐hard due to the combinatorial explosion of possible scaffold arrangements. ALLMAPS addresses this challenge by reformulating the scaffold OO problem into a Traveling Salesman Problem (TSP), a well‐known optimization problem where the goal is to find the shortest possible route that visits a set of locations and returns to the origin point. By casting scaffold OO in terms of TSP, ALLMAPS employs a Genetic Algorithm (GA) to refine scaffold OO, which is an adaptive heuristic search algorithm premised on the ideas of natural selection and genetics [24]. They are particularly well‐suited for solving complex optimization problems where traditional methods are inefficient. In the context of ALLMAPS, the GA iteratively searches for the most fitting arrangement of scaffolds that aligns with the input mapping data, effectively “evolving” toward an optimal solution over successive generations [24].

### Genome‐build quality control via *K*‐mer spectra and linkage map analyses

*K*‐mer spectra extracted from sequencing data are modeled according to two different statistical models: the negative binomial mixture model (default model) and ALLPATHS model, as described in Gnerre et al. [46]. Briefly, in the negative binomial mixture model, the *K*‐mer spectrum is decomposed into subspectra generated by parts of the genomes with different copy numbers. Formally, we want to infer *G*_*c*_ where *c* is the number of *K*‐mers with copy number *c* in the genome. The observed *K*‐mer spectrum is modeled with the mixture of negative binomial distributions and can be calculated as

$$K_{j}=\sum_{c}G_{c}\times \mathrm{Negative}\;\mathrm{Binomial}(n\times j,\;p).$$

Mixing rates $G_{c}$, shape parameters n, and p of the negative binomial distributions are estimated using numerical methods to iteratively minimize the *L*_2_ distance between the observed *K*‐mer spectrum and the generative model until convergence. The ALLPATHS model models *K*‐mers as simply categorized as haploid, diploid, and repeats [46], which is less suited for polyploid genomes and is less computationally intensive.

Linkage map analysis is carried out by computing the linkage disequilibrium (*r* ^2^) between all pairs of genetic markers along the genome

$$r^{2}=\frac{{\left(p_{\mathrm{ab}}-p_{a}p_{b}\right)}^{2}}{p_{a}\left(1-p_{a}\right)p_{b}(1-p_{b})}.$$

### Pedigree and genome diversity

The pedigree of breeding varieties is extracted from the PED file format [23]. The inbreeding coefficient for each variety is estimated by simulation of *N* (default: 10,000) alleles from progenitors, with a user‐specified ploidy level (default: 2, but with arbitrary ploidy supported). The inbreeding coefficients, *F*, are then estimated by the proportion of alleles that appear >1 time, for example, identical by descent. Copy number variations between varieties are analyzed using Mosdepth [47] to compute mean depth per genome bin from the respective BAM files and then compared side‐by‐side.

### Library dependencies

The JCVI library supports Python 3.7+ and depends on the following third‐party libraries: CrossMap, PyPDF2, Biopython, boto3, brewer2mpl, deap, ete3, ftpretty, genomepy, gffutils, goatools, graphviz, jinja2, matplotlib, more‐itertools, natsort, networkx, numpy, ortools, pybedtools, rich, scikit‐image, scipy, seaborn, and webcolors.

## AUTHOR CONTRIBUTIONS

Initial idea and conceptualization by Haibao Tang and Xingtan Zhang. All authors contributed to the development of the software. Haibao Tang, Vivek Krishnakumar, Xiaofei Zeng, Zhougeng Xu, Adam Taranto, and Johnathan S. Lomas implemented the methods and maintained the software. Yibin Wang, Yixing Zhang, and Yumin Huang performed workflow analyses. Haibao Tang, Won Cheol Yim, Jisen Zhang, and Xingtan Zhang wrote the paper. All authors have read the manuscript and approved it for publication.

## CONFLICT OF INTEREST STATEMENT

The authors declare no conflict of interest.

## ETHICS STATEMENT

No animals or humans were involved in this study.

## Data Availability

The JCVI library is publicly available on GitHub at https://github.com/tanghaibao/jcvi. Supplementary materials (graphical abstract, slides, videos, Chinese translated version, and updated materials) may be found in the online DOI or iMeta Science http://www.imeta.science/.
